# On the Absorption of Dentine; Its Relation to the Process of Reimplantation and to Decay of the Teeth

**Published:** 1887-01

**Authors:** 


### ON THE ABSORPTION OF DENTINE; ITS RELATION TO THE PROCESS OF REIMPLANTATION AND TO DECAY OF THE TEETH.

BY PROF. MILLER.

Absorption of the roots of temporary teeth has been thoroughly and carefully studied, and I have made no attempt to repeat the investigations which have been made concerning the nature of this process.

It appeared to me, however, that a study of the phenomena of absorption of dentine under conditions which exclude the possibility of the dentine itself taking any active part in the process might add something to our knowledge of this question, and possibly be of some use in determining the relation which this process bears to the fixation of reimplanted or transplanted teeth, and to decay of the teeth.

The experiments were performed upon rabbits; the material, taken from the roots of teeth partly dry and partly fresh, was sawed into sections about one millimeter thick; they were then washed in distilled water with 0,75 per cent. common salt. The dry sections were previously sterilized with absolute alcohol,. but the fresh ones were not, the aim being to keep the pericementum as unchanged as possible. The rabbit was then placed upon its back, the hair removed from the abdomen for a space as large as a silver quarter of a dollar, the skin wiped with sublimate (1-1000), a slit about one-fourth of an inch long made through the wall of the abdomen, and two or three of the sections brought into the abdominal cavity with sterilized pliers. The cut was closed by a simple stitch and protected by a coating of iodoform-collodion. Six rabbits in all were treated in this way, and apparently suffered no inconvenience whatever from the operation. The rabbits were killed after periods varying from three weeks to three months.

In three weeks the pieces were found surrounded by a thick capsule of connective tissue, densely filled with round cells. The dentine was firmly enclosed and held in the capsule, but not attached to it. On decalcifying and making sections, the dentine separated from the connective tissue, leaving a hole in each section exactly corresponding to the size and shape of the cut of dentine.

In six weeks resorption had begun at nearly all points destitute of pericementum, whereas at those points covered by pericementum a perfectly firm union had taken place with the surrounding tissue, so that the dentine could not be removed without tearing the tissue. In this case, when sections were made, the dentine did not separate from the tissue of the capsule.

In no case did a firm attachment take place where the pericementum was not present, although a slight *pseudo*-attachment was produced by the bundles of connective tissue which filled up the irregular resorption spaces. It might be put down as a law for those who practice replanting and transplanting teeth : "Preserve the pericementum, for to that extent to which it is removed, to the same will the success and permanency of the operation be hazarded." For the same reason I am inclined to think that removing the point of the root, except it be diseased, is a mistake, and will result in resorption, advancing from the apex toward the base of the root. I have read many reports of teeth being replanted after they had been out of the mouth for hours, or even days, and in some cases lain in the dirt, and which became firmly attached again.

It is quite possible that any tooth may become fixed by a sort of encapsulation of the root, or by a pseudo-attachment, as mentioned above, but such a fixation can hardly be permanent, and I think experience will show that such teeth fall out in the course of a few weeks or months. It must be borne in mind that these conclusions are based solely upon the above experiments upon rabbits. They are, however, completely confirmed by the replantation experiments of Leo Fredel,* made on dogs; also by the experiments of Morgenstern, and by my own examination of a tooth which was re-extracted two years after reimplantation.

The fixation of reimplanted or transplanted teeth may, therefore, be accomplished in three ways :

1. By simple encapsulation of the root. This mode of attachment is, I believe, frequently referred to in dental literature.

2. By the bundles of connective tissue which fill up the irregular absorption spaces, just as a very porous body might become attached to soft tissue by the latter growing into and through the interspaces. I have a number of specimens showing this mode of union, which I have called a pseudo-attachment.

3. By a direct union of the surrounding tissues with the *living* pericementum. This I am inclined to look upon as the only permanent attachment.

* Oesterreichisch-Ungarische Vierteljahrschrift, July, 1886.

In all the cases the resorption was characterized by the fewness of osteoclasts, the resorption apparently being carried on by the small round cells which accumulated in immense numbers around each piece of dentine, so that in sections stained with picro-lithio-carmine the dentine appeared as a yellow spot surrounded by a deep red border. The opinion has been expressed that in this case the resorption was produced by osteoclasts which afterwards disappeared, giving place to fibrous tissue. The absorption took place in territories, giving rise to a ragged, scalloped border, such as is ordinarily met with in roots undergoing absorption. At the end of the third month only a trace of the dentine was to be found.

It seems scarcely necessary to state that there was not a trace of a similarity between the process of absorption, as seen in the specimens under consideration, and decay of the teeth, and indeed it appears to me exceedingly doubtful whether we may, under any circumstances at all, liken absorption to *caries dentium.*

The first process takes place only in the presence of, and under the action of, living tissue; the second process is carried on completely outside of an enveloping living tissue, and only under the action of certain external agents. In the first process we find a simultaneous disappearance of the organic and inorganic constituents of the dentine, giving rise to a hard, rough, ragged surface; in the second, the destruction of the non-organic constituents goes far in advance, leaving a soft mass, as unlike the surface of a root in absorption as it possibly could be. Nor is there anything in the advanced stages of caries which could for an instant be mistaken for absorption. As for the medullary elements, claimed by a few to appear in decaying dentine, we should use a much more positive and less negative evidence than has been thus far presented before we should be justified in accepting their existence.

It is usually stated that in absorption of dentine the lime-salts are first removed, the organic matter later. I do not know on what grounds such a statement is based. It is patent to every dentist that roots undergoing absorption do not become decalcified, but that the whole tissue disappears simultaneously. In the experiments under consideration, I was not able to detect a trace of decalcification, and I have furthermore found that pieces of dentine which have been reduced to the size of a very small pinhead by absorption still remain perfectly hard and undecalcified.

